# A Spectroscopic Study on Secondary Structure and Thermal Unfolding of the Plant Toxin Gelonin Confirms Some Typical Structural Characteristics and Unravels the Sequence of Thermal Unfolding Events

**DOI:** 10.3390/toxins11090483

**Published:** 2019-08-22

**Authors:** Andrea Scirè, Fabio Tanfani, Alessio Ausili

**Affiliations:** 1 Dipartimento di Scienze della Vita e dell’Ambiente, Università Politecnica delle Marche, 60131 Ancona, Italy; 2 Departamento de Bioquímica y Biología Molecular “A”, Facultad de Veterinaria, Regional Campus of International Excellence “Campus Mare Nostrum”, Universidad de Murcia, 30100 Murcia, Spain

**Keywords:** Gelonin, Ribosome-inactivating protein, Infrared spectroscopy, Thermal unfolding, Two-dimensional correlation spectroscopy, Immunotoxins

## Abstract

Gelonin from the Indian plant *Gelonium multiflorum* belongs to the type I ribosome-inactivating proteins (RIPs). Like other members of RIPs, this toxin glycoprotein inhibits protein synthesis of eukaryotic cells; hence, it is largely used in the construction of immunotoxins composed of cell-targeted antibodies. Lysosomal degradation is one of the main issues in targeted tumor therapies, especially for type I RIP-based toxins, as they lack the translocation domains. The result is an attenuated cytosolic delivery and a decrease of the antitumor efficacy of these plant-derived toxins; therefore, strategies to permit their release from endosomal vesicles or modifications of the toxins to make them resistant to degradation are necessary to improve their efficacy. Using infrared spectroscopy, we thoroughly analyzed both the secondary structure and the thermal unfolding of gelonin. Moreover, by the combination of two-dimensional correlation spectroscopy and phase diagram method, it was possible to deduce the sequence of events during the unfolding, confirming the typical characteristic of the RIP members to denature in two steps, as a sequential loss of tertiary and secondary structure was detected at 58 °C and at 65 °C, respectively. Additionally, some discrepancies in the unfolding process between gelonin and saporin-S6, another type I RIP protein, were detected.

## 1. Introduction

Gelonin was isolated from the seeds of the Indian plant *Gelonium multiflorum*, and its biochemical/biophysics characteristics have been broadly studied, principally for its several important applications in biomedicine. Indeed, gelonin shows a strong antitumor effect in vitro in cell lines of leukemia [1], melanoma [2], breast cancer [3,4], CD80/CD86 tumors, and Hodgkin lymphoma [5]. Moreover, gelonin is also effective against the viruses of HIV-1 [6,7,8], HSV, and HHV8 [9,10], having the characteristic of being non-toxic to uninfected target cells, spermatocytes, or intact animals [7,11,12]. Gelonin is a toxin glycoprotein belonging to the type I group of ribosome-inactivating protein (RIP) family. Similar to other RIPs, the toxicity of gelonin is due to its capability of depurinating RNA in ribosomes, inhibiting irreversibly the protein synthesis of eukaryotic cells, using its high specific N-glycosidase activity on the 28S rRNA [13,14]. The members of this family are composed of only one polypeptide chain, the catalytic A-chain [15], and it is supposed that the cellular uptake of gelonin occurs via pinocytosis and mannose receptor-mediated endocytosis [16]. The absence of a binding domain, the B-chain, leads to a different intracellular distribution of gelonin compared to type II RIPs, which consist of both an A-chain and a B-chain [17]. For this reason, gelonin is thought to localize in the endosomal/lysosomal compartment where it cannot perform its toxicity to the fullest in intact cells [18]. In cell-free systems, however, gelonin shows a strong inactivation of the large ribosomal subunits, depurinating a specific adenine base A4324 from a vital region of the 28S rRNA unit [19,20].

In the past four decades, with the advent of antibody therapy, conjugates containing RIPs linked to monoclonal antibodies have been applied as immunotoxins in targeted tumor therapies [21,22]. Once internalized, the targeted toxins are delivered into early endosomes where they are destined to be recycled [23] or transported into late endosomes, and finally lysosomes for degradation. Lysosomal degradation is one of the main problems in targeted tumor therapies [24]. Since plant-derived toxins like gelonin and saporin do not include translocation domains, the cytosolic delivery of type I RIP-based targeted toxins is attenuated. Several strategies to facilitate the escape of targeted toxins from endosomal vesicles have been tested, between them there are photochemical internalization [25], cell penetration by protein transduction domains [26], or the use of lysosomotropic agents like chloroquine [27], all these techniques prevent the lysosomal degradation of targeted toxins by facilitating their endosomal escape into the cytosol, thus enhancing the anti-tumor efficacy of the targeted toxin. Besides these, the modification of targeted toxins to make them resistant to lysosomal degradation is another valuable strategy to increase the efficiency of targeted toxins [28]. Moreover, the connection between function and physicochemical properties of any potential medicament is often critical when developing the drug. A promising drug for its application, including immunotherapy, can often be ineffective due to problems, such as protein aggregation or stability during the storage. One of the most common strategies for overcoming these problems is the PEGylation of the protein, which has, however, been shown to interfere unpredictably in both immunity and safety [29,30]. Therefore, in specific cases, it could be of fundamental help to understand the structural and the stability characteristics, to select a potential candidate with the best properties that can prevent protein aggregation and that is stable without having to operate with subsequent modifications.

In this study, we investigated in detail the secondary structure of gelonin by using transmittance FTIR spectroscopy and comparing our data both to the X-ray structure and to other data obtained by circular dichroism and Raman spectroscopy. Besides, a deep insight into the thermal stability of gelonin combining two-dimensional correlation spectroscopy (2D-COS) and phase diagram analysis allowed us to detect the sequence of events in the unfolding process and to observe some differences with saporin-S6, another type 1 RIP, previously studied by our group.

## 2. Results and Discussion

### 2.1. Analysis of Gelonin Secondary Structure

**Figure 1:**
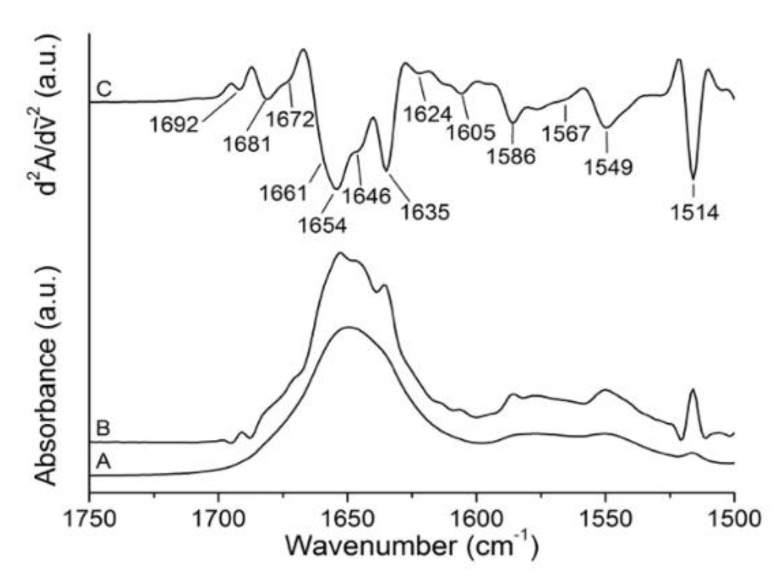
Infrared spectra of gelonin in ^2^H_2_O medium at 20 °C. A, B, and C represent absorbance, deconvoluted, and second derivative spectra, respectively.

Figure 1 displays the original absorbance (A) and the resolution-enhanced spectra (deconvoluted (B) and second derivative (C)) of gelonin at the temperature of 20 °C in the spectral range that includes both the amide I’ (1700–1620 cm^−1^) and the residual amide II (1580–1510 cm^−1^) band. From the analysis of the former, the information on protein secondary structure could be obtained, while the latter is particularly sensitive to ^1^H/^2^H exchange and is indicative of the accessibility of the protein to the solvent. The resolution-enhanced spectra of amide I’ band region showed eight component bands, and each of them could be attributed to a particular secondary structure element [31,32]. In particular, the evident peak at 1654 cm^−1^ was due to α -helix, while β-structures showed three characteristic bands arising at 1624 (low-frequency β-sheet), 1635, and 1681 cm^−1^ (high frequency β-sheet) [33,34] depending on the strength of hydrogen bonds or the coupling bands of the transition dipole formed between the strands [35]. Most probably, the low-frequency β-sheet was mainly due to the absorbance of the antiparallel β-sheet at the C-terminal domain that is particularly exposed to the solvent [32], whereas the band at 1681 cm^−1^ could be due to the contribution of β-turns that absorb in this spectral region [36]. Other bands that might be assigned to β-turns elements arose at 1661, 1672, and 1692 cm^−1^. Finally, the band close to 1646 cm^−1^ reflected the presence of unordered structures, and the contribution of 3_10_-helices overlapped the β-turns band at 1661 cm^−1^ [37]. The remaining bands displayed in Figure 1 outside the amide I’ band spectral range were related to the absorption of the lateral chains of amino acids, excluding the residual amide II band that arose near 1550 cm^−1^. In particular, the amino acids that absorbed at 1605, 1586, 1567, and 1514 cm^−1^ were arginine, aspartic acid, glutamic acid, and tyrosine, respectively.

**Figure 2:**
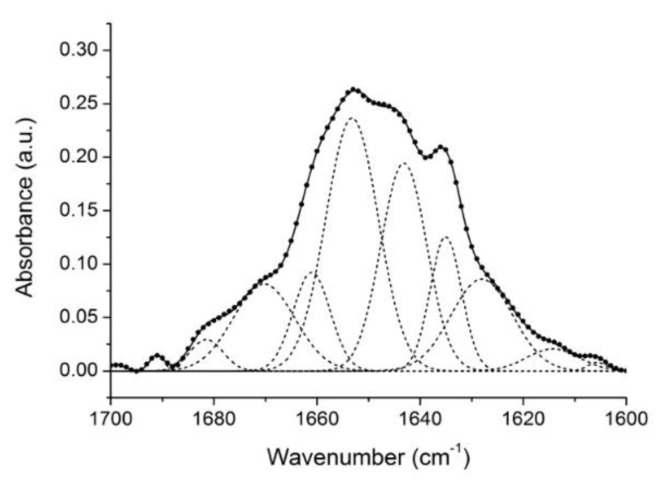
Deconvoluted amide I’ band contour (continuous line) with the best fitted Gaussian individual component bands (dashed lines). The dotted line is the original self-deconvoluted amide I’ band. All the numerical values are given in Table 1.

Gelonin has a molecular weight of 30 kDa with a primary structure of 258 amino acidic residues. It has already been crystallized, and the high-resolution three-dimensional structure has been determined (PDB code: 3KTZ); it shows the characteristic type 1 RIP motif [8,38]. Structurally, this toxin protein belongs to the (α + β) class of proteins, showing a mainly beta secondary structure N-terminal domain, while the helical structure is predominant at the C-terminal domain. The N-terminal domain is formed by six strands: Four central antiparallel and two border parallel strands linked with each other by two α-helices and one 3_10_-helix. On the other hand, six α-helices are linked by two antiparallel β-sheets and two 3_10_-helices form the C-terminal domain [38]. The protein secondary structure was also studied using other biophysical methods, such as circular dichroism and Raman spectroscopy [39,40]. We compared the secondary structure of the protein in solution at 20 °C deriving from FTIR spectra analysis to previous structural data. The deconvoluted amide I’ band decomposed into different component bands, as described above, and the curve fitting was performed by using Gaussian curves (Figure 2). The secondary structure percentage composition was estimated according to the component band area calculation, and the results are reported in detail in Table 1. From our estimation, gelonin showed a prevalent content of α-helix structures from a minimum of 32% to a maximum of 40% (including 3_10_-helices). The β-sheet elements were around 24% of the protein secondary structure, while the content of β-turns was included between 13 and 23%. Finally, the unordered structure was calculated as 22%. The fluctuations of the values were due to the assignment uncertainty of some bands.

These results were in a good agreement both with the high-resolution three-dimensional structure obtained by X-ray crystallography and with the previous estimations of a secondary structure estimated from circular dichroism and Raman spectroscopy data. Table 2 reports a comparison of the secondary structure composition calculated by the different methods. It could be noted that FTIR results were rather consistent with X-ray data, apart from the impossibility to discriminate turns and bend structures by infrared spectroscopy. Otherwise, some small differences were detected when compared to Raman spectroscopy, mainly for the higher content of turns calculated from Raman spectra, while the secondary structure analysis of the protein by circular dichroism was limited to the α-helix contribution estimation that was in line with the other data anyway.

### 2.2. Thermal Unfolding

**Figure 3:**
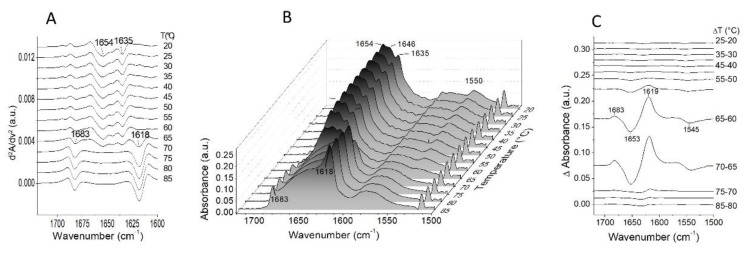
All the second derivative (**A**), deconvoluted (**B**), and difference spectra (**C**) in the range of temperature between 20 and 85 °C are reported.

To study the structural changes that occur during the thermal unfolding of gelonin, the protein was heated from 20 to 85 °C, and FTIR spectra were recorded each at 5 °C. All the second derivative and deconvoluted spectra at different temperatures are shown in Figure 3A,B, respectively. Both panels show how the protein secondary structure changed with an increase in the temperature. The resolution-enhanced bands, corresponding to the different structures, remained unaltered up to 55 °C. From this temperature, the protein began to progressively lose its secondary structure up to 70 °C, the temperature at which the gelonin was completely unfolded and aggregated. The unfolded state of the protein was reflected by the absence of the characteristic peaks due to the different secondary structures, such as the α-helix peak at 1654 cm^−1^ and β-sheet peak at 1635 cm^−1^, while the aggregation, caused by intermolecular interaction formation [41], was deduced by the two evident peaks that arose at 1683 and 1618 cm^−1^. The aggregation also indicates an irreversible denaturation process for the protein. Information on conformational changes as a function of temperature could also be obtained by the analysis of the residual amide II band at 1550 cm^−1^ (see Figure 3B). When the protein conformation became more accessible to the solvent due to the relaxation of the tertiary structure, a further ^1^H/^2^H exchange occurred, and the residual amide II band decreased until it disappeared with the ^1^H/^2^H exchange. Gelonin tertiary structure showed a gradual relaxation up to 55 °C when the protein started to undergo an evident conformational change corresponding to the secondary structure denaturation process. At 70 °C, the residual amide II band was fully disappeared. For better visualization of the structural and conformational variations that occur when the temperature was increased from 20 to 85 °C, the difference absorbance spectra were plotted (Figure 3C). These spectra were obtained by subtracting each spectrum from the previous one recorded at 5 °C lower temperature. The negative peaks at 1653 and 1545 cm^−1^ indicated the loss of protein secondary and tertiary structure, respectively, while the positive peaks at 1683 and 1619 cm^−1^ corresponded to the gelonin aggregation. The different spectra confirmed that the main structural variations occurred from 60 to 70 °C. In this temperature range, both negative and positive peaks were marked. However, some slight peaks could also be observed at higher and lower temperatures, indicating that small structural changes also occur at other temperature ranges.

**Figure 4:**
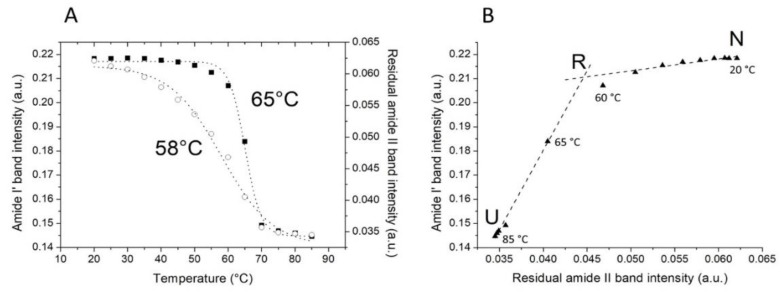
The plot of the amide I’ and residual amide II band intensities (**A**) and phase diagram representing the thermal unfolding process of gelonin (**B**). (**A**) Monitoring the intensity of the amide I’ (●) and the residual amide II (○) bands vs. temperature. The dotted lines depict the sigmoid fit based on the experimental points. (**B**) Each line represents an all-or-none transition between two states indicated as N (native), R (relaxed), and U (unfolded). Temperature values are indicated in the vicinity of the I(ν1) vs. I(ν2) points (▲).

By the analysis of the denaturation curves (Figure 4A), we could get a more overall vision of the protein structural behavior during the thermal unfolding and estimate the temperature of melting (T_m_) and the temperature of half deuteration (T_D1/2_) [42]. Here, gelonin T_m_ was calculated monitoring the amide I’ band intensity vs. temperature, while by plotting the intensity of residual amide II band as a function of the temperature, information on ^1^H/^2^H exchange and the estimation of T_D1/2_ were obtained. Both plots were best fitted by sigmoidal curves. The onset temperatures (defined as the temperature at which 10% of the band is decreased) were assessed at 58 and 41 °C for the secondary structure denaturation and ^1^H/^2^H exchange, respectively, while T_m_ was determined at 65 °C and T_D1/2_ at 58 °C. These values match quite well with previous results [40] and are indicative about the different stability of the secondary and tertiary structure of gelonin when the temperature is increased, revealing that the relaxation of the tertiary structure starts at a very low temperature compared to the beginning of the secondary structure denaturation. Moreover, T_m_ and T_D1/2_ are also quite different and indicate a sequential loss of tertiary and secondary structure [43]. The sequence of these two events has also been clearly shown by the phase diagram reported in Figure 4B. In this panel, the amide I’ and the residual amide II band intensities were plotted, and the existence of a two linear segment graph means the presence of two thermal unfolding steps from the native state (N) to unfolded state (U). The breaking point of the straight line represents an intermediate conformation of the protein that corresponds to a tertiary structure relaxation (R) with no evident changes of the secondary structure. This state is very habitual in RIPs, for example, it was already observed in saporin-S6, ricin, trichosanthin, α- and, β-momorcharin [44,45,46,47,48,49], but not in gelonin. However, the interpretation of this intermediate is uncertain, some authors described it as a molten globule-like state [50], while others tended to exclude this hypothesis and limited the interpretation of this phenomenon as a relaxation of the protein tertiary structure. Our team already observed this intermediate in saporin-S6 by FTIR, and we excluded the existence of a stable intermediate state during the thermal unfolding [49]. The absence of the typical infrared spectral characteristics of the molten globule-like state [49,51], also in gelonin spectra, made us conclude that even in this case we cannot speculate on the existence of this thermodynamic state. Here, as it was described for saporin-S6, there is an evident non-cooperative unfolding process that occurs in two steps: the first one from native to a conformational relaxed state, and the second step from the relaxed to the unfolded state. In this case, it is also worth noting that, in this two-step process, while the first step is potentially reversible as it is a relaxation of the tertiary structure, the second step is completely irreversible since the protein aggregates. Therefore, the whole process can be summarized as Native state ↔ Relaxed state → Unfolded/Aggregated state. In such a model, the aggregation can shift the equilibrium in one direction or the other and can depend on both the sample concentration and the temperature scan rate. In this work, for experimental reasons, the protein concentration was 50 mg/ml, and the scan rate was 0.5 °C/min, and even if the aim was not to carry out a thermodynamic or kinetic study of the gelonin unfolding, it is important to consider these parameters and observations for a rational interpretation of the results.

### 2.3. The Sequence of the Thermal Unfolding Events

**Figure 5:**
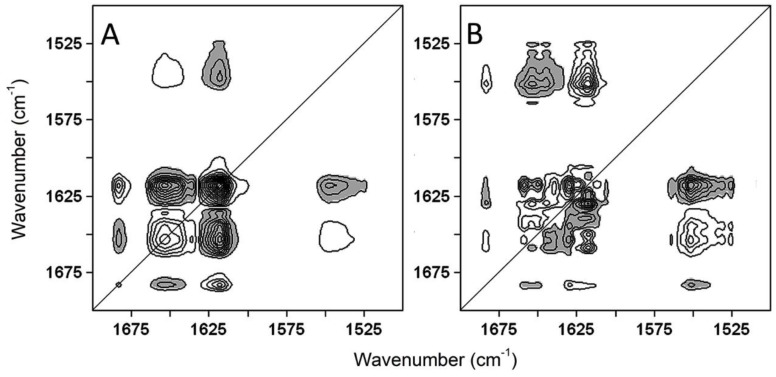
2D-COS (two-dimensional correlation spectroscopy) plots of gelonin in ^2^H_2_O during heating between 20 and 85 °C. Plots (**A**) and (**B**) represent the synchronous and the asynchronous spectra, respectively. The positive peaks are displayed in white, while the negative peaks are shown in grey. The intensity of the peaks is represented by the multiple lines.

Gelonin denaturation process was investigated in more detail using two-dimensional correlation spectroscopy. The 2D-COS spectra were analyzed between 1700 and 1500 cm^−1^ from 20 to 85 °C. Figure 5 shows both the synchronous (Figure 5A) and asynchronous (Figure 5B) maps: positive and negative peaks are displayed, respectively, in white and grey color, and the contour line number is directly proportional to the peak intensity. The 2D-COS analysis was performed examining the upper triangle of the maps, being ν_1_ and ν_2_, respectively, abscissa and ordinate axis, and following the rules described by Noda [52].

From the synchronous analysis, the three auto-peaks at 1683, 1654, and 1618 cm^−1^ represented the main events that occurred during the denaturation process of gelonin, that are the unfolding of α-helix structures and the formation of intermolecular interactions as a consequence of the thermal unfolding. The auto-peaks corresponding to the ^1^H/^2^H further exchanged, and β-sheet unfolding could also be detected but at lower ranges of temperature and using a lower detection threshold, respectively (data not shown). In the synchronous map (Figure 5A and Table 3), there were seven cross-peaks, indicating which of these peaks would increase or decrease with an increase in the temperature, and from the correlation between peaks and secondary structures, it is possible to determine both the unfolding of some structures and the formation of others. In particular, the decrease of the peaks centered at 1550, 1635, 1654 cm^−1^ was associated with the gelonin tertiary structure relaxation (band at 1550 cm^−1^) and the secondary structure denaturation (1635 and 1654 cm^−1^), while the arising of the bands at 1618 and 1683 cm^−1^ was due to the protein aggregation. Combining this information with the analysis of the asynchronous map, we could obtain the sequential order of the events that occur during the unfolding of the protein. In the case of gelonin, the main asynchronous peaks (shown in Figure 5B and Table 3) arose roughly at the same positions of the synchronous ones and described the progressive order of the events associated with each peak. Consequently, a comprehensive analysis of the gelonin 2D-COS spectra (resumed in Table 3) gave us the complete sequence of events: The relaxation of the tertiary structure (1550↓) was the first event that happened when the temperature was rising, followed by the denaturation of the protein, first 3_10_-helix structures (1661↓), then α-helix, and β-sheet (1654↓, 1624↓, 1635↓), and finally β-turns (1672↓). After the loss of the secondary structure, gelonin underwent aggregation (1618↑, 1683↑) caused by the formation of the intermolecular interaction as a consequence of the denaturation. Finally, the complete sequence of events occurred during the thermal unfolding of gelonin is resumed in Table 4.

Combining 2D-COS results with the analysis of linear FTIR spectra and phase diagram, it is evident that the protein denaturation is an overall non-cooperative process; nevertheless, the two steps that were identified did not correspond to different thermodynamic states of the protein, but non-simultaneous changes in tertiary and secondary structure. This behavior is common in all RIPs [44,45,46,47,48,49], but gelonin showed some interesting particulars if compared to saporin-S6 that was already studied in detail with the same methods [49]. Indeed, despite the two proteins are member of the same family and present analogous structural and stability characteristics, they also show important dissimilarities. The two proteins showed a highly similar three-dimensional structure as seen from the comparison of the resolved crystal structures, with a calculated secondary structure of 37% α-helix, 24% β-sheet, 18% β-turns, and 21% unordered in the case of gelonin and 38% α-helix, 23% β-sheet, 12% β-turns, and 27% unordered in the case of saporin-S6. Even when comparing the structural data of the proteins in aqueous solution, a clear similarity was observed both with the X-ray data and between the two proteins [49]. Moreover, both proteins showed a remarkable resistance to the thermal denaturation, but gelonin exhibited a T_m_ 8 °C higher than saporin-S6 as well as a difference of 4 °C in T_D1/2_. Moreover, while in the sequence of events of saporin-S6, the first step was characterized by a slight loss of secondary structure and the beginning of protein aggregation concomitantly with the tertiary structure relaxation, in the case of gelonin, it is not possible to associate any structural change to the conformational relaxation that occur during the first step. It is also worth noting, in gelonin, the absence of the sequentiality between α-helix and β-sheet structures denaturation. On the contrary, in the case of saporin-S6, the higher thermostability of the predominately β-sheet N-terminal domain than the α-helix-rich C-terminal indicated higher flexibility of the C-terminal region and confirmed its importance in the interaction with the ribosome [49,53]. Consequently, it is not possible to deduce which domain of gelonin is more flexible or rigid. One explanation for this diverse behavior of the two proteins in solution could lie in the small structural differences, in particular, in the higher presence of the less stable α-helix structures in saporin-S6 and of the more stable β-turns in gelonin.

## 3. Conclusions

In the last decades, several strategies for the escaping of toxins from endosomal vesicles or for the modification of the toxin itself to make it less prone to lysosome degradation have been studied. In this perspective, further information about protein toxins from plants and bacteria are necessary for the development of new therapeutic approaches. In the present paper, we described in detail the unfolding process of gelonin, a type I RIP family member, using FTIR spectroscopy. The protein secondary structure was analyzed and compared to previous data obtained by different techniques and in different conditions, such as X-ray, circular dichroism, and Raman spectroscopy. The diverse secondary structure analysis showed a good similarity; in particular, X-ray and FTIR spectroscopy results were roughly coincident. Besides, a meticulous study of the behavior of gelonin at increasing temperatures, including phase diagram and 2D-COS analysis, allowed us to confirm the existence of two states in the unfolding process for gelonin. Through these analyses, we also revealed the unfolding sequence of events, from which it was possible to highlight some differences between gelonin and saporin-S6, another type I RIP previously studied by the same authors.

## 4. Materials and Methods

### 4.1. Materials

Deuterium oxide (99.9% ^2^H20), ^2^HCl, NaO^2^H, Na_2_HPO_4_, and NaH_2_-PO_4_ were purchased from Sigma-Aldrich (Milan, Italy). All other reagents and solvents were commercial samples of the highest purity.

Gelonin was purified from *Gelonium multiflorum* seeds, following previously described procedures [54].

### 4.2. FTIR Spectra

Gelonin was analyzed in 50 mM sodium phosphate ^2^H_2_O buffer, p^2^H 7.4. The p^2^H corresponds to the pH meter reading +0.4 [55]. About 1.5 mg of protein was concentrated into an approximate volume of 30 μL by using an Amicon Ultra-0.5 Centrifugal Filter with Ultracel-10 membrane (Millipore, Bedford, MA, USA) and centrifuging at 10,000 g at 4 °C. Then, the protein was washed 5 times to fully exchange the original buffer, adding 200 μL of the 50 mM sodium phosphate ^2^H_2_O buffer and re-concentrating. Finally, the sample was incubated O/N, the volume was reduced to about 30 μL, and it was placed directly between two CaF_2_ windows separated by a 25 μm Teflon spacer and assembled in a thermostated GS20500 cell (Graseby-Specac Ltd., Orpington, Kent, UK).

Measurements were performed on a Perkin-Elmer 1760-x Fourier transform infrared spectrometer (PerkinElmer, Inc., Waltham, MA, USA) equipped with a deuterated triglycine sulfate (DTGS) detector and a normal Beer-Norton apodization function. Typically, 32 scans for each background and sample were recorded, and the spectra were obtained with a nominal resolution of 2 cm^−1^. During the experiment, the spectrometer was completely purged with dry air. Sample and buffer spectra were collected by heating from 20 to 85 °C at intervals of 5 °C and 6 min delay between each scan. The time required to acquire a single scan was approximatively 4 min, resulting in a scan rate of about 0.5 °C/min. Spectra were recorded and processed using the Spectrum software from Perkin-Elmer (Version 2.1.0, PerkinElmer, Inc., Waltham, MA, USA). The buffer contribution was subtracted, as previously described [56,57]. Second derivative spectra were calculated over a 9 data-point range (9 cm^−1^), and the parameters of the deconvoluted spectra were set with a γ value of 2.5 and smoothing length of 60 [58]. Different spectra were obtained by subtracting the spectrum recorded at the lower temperature from the one recorded at 5 °C higher [59]. The estimation of gelonin secondary structure composition was performed by curve fitting of the amide I’ band [32,60] using the peak fitting module of the OriginPro software (Version 8.5.0, OriginLab Corporation, Northampton, MA, USA). The band shape for the component bands was set to a Gaussian curve, and the fitting was obtained by iteration in two steps, as described earlier [61,62]. The percentage of each secondary structure element was determined by integrating each component band obtained from the curve fitting and expressing the value as a proportion of the total amide I’ band area. To calculate the midpoint transitions, namely the temperatures of melting (T_m_) and the temperatures of half deuteration (T_D1/2_), different parameters extrapolated from the FTIR spectra of the samples were plotted against the temperature, and the raw data were fitted with a sigmoid function, as described in [63].

### 4.3. Phase Diagram Method

Infrared spectra were analyzed by phase diagram method to detect possible protein unfolding intermediates [64,65]. Indeed, this approach, based on the graphical association of different spectral intensity values (I(ν_1_) vs. I(ν_2_)) can reveal if, during the unfolding/refolding process, a protein undergoes conformational modifications characteristic of intermediates like molten globule, quaternary structure changes, or cooperative nature of the process. In our case, I(ν_1_) vs. I(ν_2_) measured at wavenumbers ν_1_ and ν_2_ of FTIR spectra, were plotted for each temperature allowing the detection of the transitions from the folded to the unfolded state of gelonin. A linear distribution of I(ν_1_) vs. I(ν_2_) represented an all-or-none transition between two thermodynamic states. Otherwise, a non-linear phase diagram would reflect a multi-state transition where each linear breaking point would represent a different state.

### 4.4. Two-Dimensional Correlation Spectroscopy (2D-COS)

Generalized 2D-COS analysis of gelonin FTIR deconvoluted spectra was performed, according to Noda [66]. The 2DShige software (Shigeaki Morita Kwansei-Gakuin University, 2004–2005) was used to create synchronous and asynchronous spectra from the dynamic spectra obtained from the temperature-induced dynamic fluctuation of spectroscopic signals. In a protein denaturation process, synchronous spectra give information about structural changes that happen simultaneously or coincidently, while the asynchronous spectra reveal the sequentiality and the non-synchronicity of the unfolding events. Combining the information obtained from both spectra, it is possible to deduce the complete sequence of the events that occur during the protein thermal denaturation [67,68].

## Figures and Tables

**Table 1:** Calculated positions (cm^−1^) and fractional areas (%) of the deconvoluted amide I’ component bands for gelonin at 20 °C.

Band Assignment	Center (cm^−1^)	Area (%)
β-sheet (low frequency)	1624	13
β-sheet	1635	10
Unordered	1646	22
α-helix	1654	32
β-turns/3_10_-helix	1661	8
β-turns	1672	12
β-turns/β-sheet (high frequency)	1681	2
β-turns	1692	1

The curve fitting value of χ^2^ was 1.1 × 10^−6^.

**Table 2:** Comparison between gelonin secondary structure composition (expressed as a percentage) calculated in this work, X-ray data [38], circular dichroism [39], and Raman spectroscopy [40].

Secondary Structure	This Work	X-ray Data	Circular Dichroism	Raman Spectroscopy
α-helix (including 3_10_-helix)	32–40	37	> 29	32
β-sheet	23–25	24		20
Turns	13–23	11		26
Bend		7		
Unordered	22	21		22

**Table 3:** Information deduced from the gelonin 2D-COS (two-dimensional correlation spectroscopy) spectra analysis in the interval of temperature between 20 and 85 °C, as shown in Figure 5.

Synchronous Map		Asynchronous Map
Auto-Peaks (cm^−1^)	Cross-Peaks at ν_1_/ν_2_ (cm^−1^)	Cross-Peaks at ν_1_/ν_2_ (cm^−1^)
1683↑	1683↑/1618↑ +	1683↑/1550↓ + a
1654↓	1683↑/1654↓ −	1683↑/1624↓ − a
1618↑	1654↓/1550↓ +	1683↑/1654↓ + a
	1654↓/1618↑ −	1661↓/1550↓ − a
	1654↓/1635↓ +	1661↓/1618↑ − b
	1635↓/1618↑ −	1654↓/1550↓ − a
	1618↑/1550↓ −	1654↓/1618↑ − b
		1654↓/1661↓ − a
		1654↓/1683↑ − b
		1624↓/1618↑ + b
		1618↑/1550↓ + a

The arrows ↑ and ↓ indicate the increase and decrease of the intensity of FTIR bands; the sign “+” represents a positive cross-peak, while the sign “−“ corresponds to a negative one; (a) and (b) mean that the variation of the peak intensity at ν_1_ (abscissa axis) happens principally after and before ν_2_ (ordinate axis), respectively.

**Table 4:** Sequence of unfolding events for gelonin as obtained by the analysis of linear FTIR spectra, phase diagram, and 2D-COS.

Unfolding Event	FTIR Band Assigment
**First step (from 20 to 60 °C)**	
Tertiary structure relaxation	(1550↓)
**Second step (from 60 to 85 °C)**	
β-turns/3_10_-helix denaturation	(1661↓)
α-helix and β-sheet denaturation	(1654↓, 1624↓, 1635↓)
Aggregation and β-turns denaturation	(1618↑, 1672↓)
Aggregation	(1683↑)

The arrows ↑ and ↓ indicate the increase and decrease of the intensity of FTIR bands.
